# Cellular and Molecular Mechanisms of Arrhythmia by Oxidative Stress

**DOI:** 10.1155/2016/9656078

**Published:** 2016-02-15

**Authors:** Ali A. Sovari

**Affiliations:** Cardiac Electrophysiology Section, Heart Institute, Cedars Sinai Medical Center, 127 S. San Vicente Boulevard, A3308, Los Angeles, CA 90048, USA

## Abstract

Current therapies for arrhythmia using ion channel blockade, catheter ablation, or an implantable cardioverter defibrillator have limitations, and it is important to search for new antiarrhythmic therapeutic targets. Both atrial fibrillation and heart failure, a condition with increased arrhythmic risk, are associated with excess amount of reactive oxygen species (ROS). There are several possible ways for ROS to induce arrhythmia. ROS can cause focal activity and reentry. ROS alter multiple cardiac ionic currents. ROS promote cardiac fibrosis and impair gap junction function, resulting in reduced myocyte coupling and facilitation of reentry. In order to design effective antioxidant drugs for treatment of arrhythmia, it is essential to explore the molecular mechanisms by which ROS exert these arrhythmic effects. Activation of Ca^2+^/CaM-dependent kinase II, c-Src tyrosine kinase, protein kinase C, and abnormal splicing of cardiac sodium channels are among the recently discovered molecular mechanisms of ROS-induced arrhythmia.

## 1. Scope of the Problem in Treatment of Arrhythmias

Cardiovascular disorders are the most common cause of death in the United States and most of the developed countries [1]. Ventricular fibrillation (VF) and ventricular tachycardia (VT) are the most common cause of sudden cardiac death (SCD) [2]. Atrial fibrillation (AF), although usually not life threatening, is associated with higher thromboembolism, increased mortality, and high healthcare cost and its incidence is increasing [3–8].

Current therapies for treatment of arrhythmias are antiarrhythmic drugs, catheter ablation, and implantable cardioverter defibrillators (ICDs) for VT/VF. Although these therapies have had some success, they have limitations. Ion channel blockade has important limitations for chronic treatment and prevention of those arrhythmias. For example, the Cardiac Arrhythmia Suppression Trial (CAST) showed that treatment of premature ventricular contractions (PVCs) with class IC antiarrhythmic drugs may increase cardiovascular mortality in patients with myocardial infarction (MI) [9]. Chronic treatment of AF with current antiarrhythmic drugs has not been more effective than a rate control strategy in reducing thromboembolism [10] which may suggest the ineffectiveness of the antiarrhythmic drugs in maintaining the sinus rhythm. Paradoxically, a common adverse effect of all currently available antiarrhythmic drugs is proarrhythmia [11, 12]. Catheter ablation therapy is based on creating an anatomically fixed lesion in the heart in order to block the reentrant circuit or propagation of the focal activity. Studies using optical mapping of the heart have shown ectopic foci and reentrant circuits are usually multiple and dynamic in complex arrhythmias such as VF and AF [13–15]. Catheter ablation for VT is primarily an adjuvant therapy for reduction of symptoms in patients with ICD [16] and cannot provide a reliable method for prevention of SCD. Defibrillation has shown success in terminating VT/VF. Nevertheless, it does not prevent the occurrence of arrhythmia, and frequent ICD shocks worsen the quality of life and may even increase mortality [16]. ICDs are relatively expensive. A study that evaluated data from multiple randomized clinical trials estimated that the cost of the ICD-related primary prevention of SCD ranged from $34,000 to $70,200 for each life-year [17]. In addition, about 70% of the patients who receive an ICD never have any appropriate defibrillation and only 30% of patients with sudden cardiac arrest (SCA) meet current criteria for implantation of ICD [18].

Many of the aforementioned limitations of current therapies for arrhythmia arise from the fact that these therapies do not address the underlying pathophysiology of arrhythmia. A more successful therapeutic approach may arise from targeting upstream pathologies that result in abnormalities in ionic currents and emergence of reentry and focal activity. Oxidative stress, which is an imbalance between production and neutralization of reactive oxygen species (ROS), is an example of a possible upstream therapeutic target. Most clinical risk factors of AF such as hypertension, age, and cardiothoracic surgeries are conditions that are associated with oxidative stress [19]. Serum markers of oxidative stress have been shown to be elevated in patients with AF [20–22]. AF in human is associated with a significant reduction in the expression of antioxidant genes as well as a significant increase in the expression of five genes related to ROS, supporting a shift toward prooxidation state in AF [23]. Cardiomyopathy, which is associated with significantly higher risk of VT/VF, is associated with oxidative stress and increased oxidation and carbonylation of proteins [24]. Perfusion of H_2_O_2_ of hearts in the Langendorff setting induces VT/VF and AF [13–15, 25], providing evidence that ROS elevation can be a cause of arrhythmia.

Despite considerable evidence that ROS play an important role in the genesis of arrhythmia, limited clinical studies using conventional antioxidants have shown conflicting results [26]. The biology of ROS is complex and designing an effective antioxidant therapy for arrhythmia requires an in-depth understanding of the cardiac sources of ROS, the ROS molecules structures and properties, triggers of ROS production, and the downstream effects of ROS which result in arrhythmia. The study of mechanisms by which ROS elevation may result in arrhythmia may lead to the discovery of novel therapeutic targets for treatment of arrhythmia.

## 2. Oxidative Stress Facilitating Focal Activity and Reentry

ROS can lead to focal activity. It has been shown that ROS prolong action potential duration (APD) in rat and guinea pig myocytes and induce early afterdepolarizations (EADs) and delayed afterdepolarization (DADs) [27]. Consistent with that finding, ROS has been shown to facilitate ventricular arrhythmia in aged and hypertensive rat hearts mainly via an EAD mechanism [25, 28].

ROS can also provide substrate for reentry. One possible mechanism for reentry in oxidative stress is via heterogeneous APD prolongation in which at a moment one area with shorter APD is excitable while the area with prolonged APD is not excitable [15, 25, 29]. In an angiotensin II activation mouse model with elevated ROS levels and in a mitochondrial oxidative stress mouse model conduction velocity (CV) is decreased and inducible VTs are mainly caused by reentry [30–32].

The results from mathematical modeling studies have supported both reentry via decreased in conduction CV and focal activity via EAD/DAD mechanisms for ROS-mediated arrhythmias [13, 33]. It is not clear in which conditions and at what levels ROS may promote one of the two mechanisms: focal activity and reentry. Nevertheless, ROS can promote both.

## 3. Arrhythmogenic Ionic Effects of Oxidative Stress

ROS elevation affects several ionic currents in cardiomyocytes. The effect of ROS on total and late Na^+^ current is important and can be arrhythmogenic. One of the mechanisms of H_2_O_2_-induced APD prolongation and EAD formation is by promoting an enhanced late sodium (Na^+^) current [34]. Ranolazine, a late Na^+^ current blocker, can suppress ROS-mediated EADs and arrhythmia, which suggests a role for the increased late Na^+^ current in the pathogenesis of ROS-mediated arrhythmia [25]. Treatments with H_2_O_2_ and angiotensin II enhance the late Na^+^ current; however, those treatments decrease the overall Na^+^ current in isolated myocytes partially through the downregulation of SCN5A transcription [35]. While an increase in late Na^+^ current may result in arrhythmia via an EAD mechanism, the reduction in total Na^+^ current seen by ROS may cause a reduction in CV and provide substrate for reentry. ROS can downregulate cardiac sodium channels and mitochondrial antioxidants can reverse this effect [36]. This can also provide a substrate for arrhythmia in a similar fashion that happens in Brugada syndrome [37–39].

ROS may also alter intracellular Ca^2+^ handling in a way that generates arrhythmia. ROS may stimulate the L-type Ca^2+^ current, which can facilitate EAD [40]. In addition, hydroxyl radicals increase the open probability of cardiac ryanodine receptors, which control the Ca^2+^ release from the sarcoplasmic reticulum (SR) to the cytoplasm [41]. Abnormal Ca^2+^ release from SR during diastole may result in formation and propagation of DADs [42, 43]. Even a brief exposure to OH^−^ significantly decreases SR Ca^2+^ uptake, which leads to an increased Ca^2+^ level in myocytes during diastole [44]. This short-term effect on Ca^2+^ transport is likely due to the OH^−^-mediated peroxidation of lipid membranes and protein sulfhydryl formation, which leads to an indirect effect on the SR Ca^2+^ transporter [44]. Increase in intracellular levels of Ca^2+^ can then translate to an inward current by sodium-calcium exchanger (NCX) action. In addition ROS may increase directly NCX activity [43].

In addition to the cardiac Na^+^ current and intracellular Ca^2+^ handling, oxidative stress affects also cardiac potassium currents. ROS may inhibit K_ATP_ channels [45] and downregulates *I*_to_ [46, 47]. These effects of ROS and its possible suppression of *I*_Kr_ and *I*_Ks_ [48] can potentially decrease the repolarization reserve, prolong the APD, and facilitate triggered activity.

In summary, ROS can affect all major ionic currents with increases in the late Na^+^ current, L-type Ca^2+^ current, leak of Ca^2+^ from SR, and NCX activity. ROS decrease peak sodium current and SERCA-mediated SR Ca^2+^ uptake. All these changes are likely to increase intracellular Ca^2+^ levels, prolong the APD, reduce of CV, and facilitate triggered activity and reentry.

## 4. Oxidative Stress and Cell Coupling

Oxidative stress promotes myocardial fibrosis [49, 50]. Biophysical properties of the extracellular matrix (ECM) are important factors that can affect the propagation of the action potential in the heart. For example, increased collagen deposition in the ECM, which is seen in increased myocardial fibrosis and scar tissue formation after myocardial infarction, may provide a barrier to the AP propagation and contribute to reentry [51]. In addition, collagen deposition may reduce electrical coupling between myocytes and facilitate focal activities by reducing the sink-to-source effect in the region of the heart [14]. Proliferation of fibroblasts and their transformation to myofibroblasts may be associated with myocyte-fibroblast coupling, which is potentially arrhythmogenic via increased ectopic activity [52].

Another important factor that affects electrical coupling of myocytes is gap junction function, and oxidative stress impairs gap junction conduction [30, 53, 54]. Gap junctions are channels in all compartments of the heart that form the conduction pathways between cells allowing for an electrical syncytium. Connexin 43 (Cx43) is the major component of gap junctions in the ventricular myocytes, and it is one of the important connexins in the atria. Cx43 is decreased in human heart failure, a condition that is associated with significantly increased ROS levels and increased risk of arrhythmia [24, 55–57]. In cardiac angiotensin II activation and mitochondrial oxidative stress mouse models, ROS elevation significantly decreases Cx43 levels, impairs gap junction conduction, and results in spontaneous and pacing induced arrhythmia [31, 32, 58, 59]. Treatments with a mitochondria-targeted antioxidant and angiotensin receptor blockers prevent the gap junctional remodeling and arrhythmias, supporting a key role for oxidative stress in Cx43 remodeling and impairing electrical coupling between myocytes [30–32].

## 5. Molecular Mechanisms of ROS-Induced Arrhythmia

While ROS seems to be able to cause arrhythmias, at least when externally supplied, and there are some possible alterations that can explain the arrhythmogenic substrate created by ROS, more effort and focus are required to explore the molecular mechanisms by which ROS result in those abnormalities. These mechanisms may involve the effect of ROS on genes, transcriptional regulation, protein trafficking, and posttranslational modifications. There are examples of studies that described novel molecular mechanisms for ROS-induced arrhythmia, which may result in discovering potentially new antiarrhythmic targets; however, there are much more research needed in this area.

The molecular mechanism by which ROS affect the cardiac Na^+^ current and how the Na^+^ current reduction can be prevented will be important steps toward designing new and effective antiarrhythmic drugs. Abnormal splicing of cardiac sodium channel mRNA is a possible mechanism for the decreased sodium current in arrhythmia. The splicing factors RBM25 and LUC7L3 are elevated in human heart failure tissue and mediate truncation of SCN5A mRNA in both Jurkat cells and human embryonic stem cell-derived cardiomyocytes [60]. RBM25/LUC7L3-mediated abnormal SCN5A mRNA splicing reduces Na^+^ channel current to a range known to cause sudden cardiac death [61]. It has been shown that angiotensin II and hypoxia, known to cause elevation in ROS levels [62–64], are associated with the aforementioned splicing factor abnormalities resulting in a Na^+^ current reduction in the heart [60]. Another mechanism by which ROS may affect cardiac sodium current is probably via protein kinase C (PKC). It has been shown that mitochondrial ROS causes a reduction in cardiac sodium current and that effect can be prevented by inhibition of PKC [36]. In addition, c-Src inhibition can recover cardiac Na^+^ current to normal in a mouse model of manganese superoxide dismutase deficient with elevated mitochondrial ROS, which suggests that c-Src partially mediates the effect of ROS on cardiac Na^+^ current reduction [59].

Ca^2+^/CaM-dependent kinase II (CaMKII) can be activated by ROS, and its activation probably mediates several of the ROS-induced arrhythmogenic effects [65]. If CaMKII is activated under prooxidant conditions, two methionine residues become oxidized, and the sustained activation of CaMKII, independent of its binding to Ca^2+^/CaM, occurs [66]. Pretreatment of hypertensive rat hearts with a CaMKII-inhibitor KN-93 prevents VT inducibility during oxidative stress [67]. These findings are supported by both patch-clamp and Ca^2+^-imaging studies, which demonstrate that the oxidative-stress-induced activation of CaMKII causes arrhythmias [68]. Several recent studies have linked the use of CaMKII inhibitors to a decrease in catecholaminergic polymorphic ventricular tachycardia [69, 70].

Other possible arrhythmogenic mechanisms by which CaMKII activation exerts arrhythmic effects include RyR phosphorylation and activation under oxidative stress [71, 72]. An increase in the open probability of RyR is thought to be mediated by CaMKII activation [42]. In addition, CaMKII has been shown to shift the voltage dependence of Na^+^ channel availability by approximately +5 mV, to hasten recovery from inactivation, and to increase late Na^+^ current in cardiomyocytes [73]. CaMKII plays an important role in L-type Ca^2+^ channel facilitation, the Ca^2+^-dependent augmentation of Ca^2+^ current (*I*_CaL_) exhibited during rapid repeated depolarization [74]. In addition, CaMKII has been identified recently as one of the mediators of fibroblast proliferation in response to angiotensin II [75]. Because CaMKII is a relatively indiscriminate kinase, it is very possible that other mechanisms are involved in the genesis of arrhythmia by CaMKII activation under oxidative stress.

## 6. Conclusion

**Figure 1:**
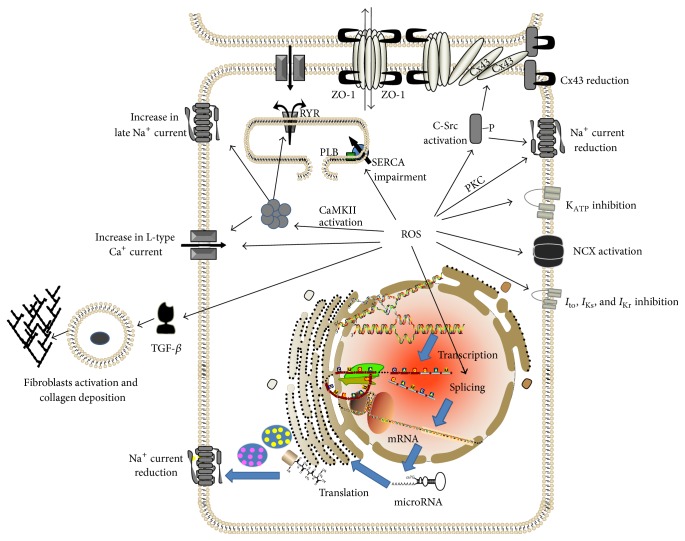
Schematic review of some of the important known mechanisms by which excess ROS may induce arrhythmia. Activation of CaMKII, c-Src, and PKC may mediate several important effects of ROS on ionic currents resulting in arrhythmia. In addition, ROS adversely affect splicing of mRNA of cardiac sodium channels resulting in abnormal truncated cardiac sodium channel proteins and a reduction in normal sodium channels. ROS also increase fibrosis and impair gap junction conduction, resulting in reduced myocyte coupling. Abnormal splicing, activation of CaMKII, c-Src, and PKC are among emerging new antiarrhythmic therapeutic targets. CaMKII: Ca^2+^/calmodulin-dependent protein kinases II; CX43: connexin 43; NCX: Na^+^/Ca^2+^ exchanger; PLB: phospholamban; ROS: reactive oxygen species; RYR: ryanodine receptor; SERCA: sarco-/endoplasmic reticulum Ca^2+^-ATPase; TGF-*β*: Transforming Growth Factor-*β*; ZO-1: Zonula Occludens-1.

Ventricular and atrial arrhythmias place considerable burden on the healthcare system. Currently, available therapies for arrhythmia have certain limitations. In order to identify new, effective therapeutic targets for treatment of arrhythmia, mechanisms of the genesis of arrhythmia should be further explored. One possible upstream therapeutic target for treatment of arrhythmia is oxidative stress. It has been shown that excess amount of ROS can result in both reentry and focal activity by modifying many of the ionic currents in cardiomyocytes, cardiomyocyte coupling, and important elements of the extracellular matrix (Figure 1 and Table 1). Molecular mechanisms by which ROS exert those effects are the key areas under investigation. Activation of CaMKII, c-Src, PKC, and abnormal splicing of cardiac sodium channels are among the emerging new therapeutic targets.

## Figures and Tables

**Table 1:** A summary of mechanisms of oxidative stress induced arrhythmia and potential therapeutic targets.

Affected ion channels	Na^+^ current reduction (via PKC and c-Src, also via abnormal splicing) ⇒ reduction in conduction velocity
	KATP inhibition ⇒ repolarization abnormality
	NCX activation ⇒ increasing inward current and facilitating afterdepolarization
	*I*_to_, *I*_Kr_, *I*_Ks_ inhibition ⇒ abnormal repolarization
	Increase in inward Ca^++^ current (direct or via CaMKII activation) ⇒ facilitating afterdepolarization
	Increase in late Na^+^current ⇒ facilitating afterdepolarization

Effect on intracellular Ca^++^ handling	Impairment of SERCA ⇒ increase intracellular Ca^++^ levels ⇒ facilitating afterdepolarization
	Affecting RyR receptor (via CaMKII activation) ⇒ leakiness of SR ⇒ increase intracellular Ca^++^ levels ⇒ facilitating afterdepolarization

Effect on myocyte-myocyte coupling	Affecting assembling of Cx43 at gap junctions ⇒ reduction in conduction velocity

Effect on extracellular matrix	Activating fibrotic process (via TGF-*β*) ⇒ reduction in conduction velocity and impaired myocyte-myocyte coupling due to collagen deposition

CaMKII: Ca^2+^/calmodulin-dependent protein kinases II; Cx43: connexin 43; NCX: Na^+^/Ca^2+^ exchanger; RyR: ryanodine receptor; SERCA: sarco-/endoplasmic reticulum Ca^++^ - ATPase; TGF-*β*: Transforming Growth Factor-*β*.

## List of Abbreviations

AF: Atrial fibrillation
APD: Action potential duration
CaMKII: Ca^2+^/calmodulin-dependent protein kinases II
Cx43: Connexin 43
CV: Conduction velocity
DAD: Delayed afterdepolarization
EAD: Early afterdepolarization
ICD: Implantable cardioverter defibrillator
NCX: Na^+^/Ca^2+^ exchanger
PLB: Phospholamban
ROS: Reactive oxygen species
RyR: Ryanodine receptor
SERCA: Sarco-/endoplasmic reticulum Ca^2+^-ATPase
SCA: Sudden cardiac arrest
SCD: Sudden cardiac death
TGF-*β*: Transforming Growth Factor-*β*
VT: Ventricular tachycardia
VF: Ventricular fibrillation
ZO-1: Zonula Occludens-1.
